# Graphene/Ruthenium Active Species Aerogel as Electrode for Supercapacitor Applications

**DOI:** 10.3390/ma11010057

**Published:** 2017-12-30

**Authors:** Arnaud Gigot, Marco Fontana, Candido Fabrizio Pirri, Paola Rivolo

**Affiliations:** 1 Center for Sustainable Future Technologies, Istituto Italiano di Tecnologia, 10129 Torino, Italy; fabrizio.pirri@polito.it; 2 Dipartimento di Scienza Applicata e Tecnologia, Politecnico di Torino, 10129 Torino, Italy; marco.fontana@polito.it (M.F.); paola.rivolo@polito.it (P.R.)

**Keywords:** reduced graphene oxide, aerogels, ruthenium sulphide, supercapacitor, hybrid nanocomposite

## Abstract

Ruthenium active species containing Ruthenium Sulphide (RuS_2_) is synthesized together with a self-assembled reduced graphene oxide (RGO) aerogel by a one-pot hydrothermal synthesis. Ruthenium Chloride and L-Cysteine are used as reactants. The hydrothermal synthesis of the innovative hybrid material occurs at 180 °C for 12 h, by using water as solvent. The structure and morphology of the hybrid material are fully characterized by Raman, XRD, XPS, FESEM and TEM. The XRD and diffraction pattern obtained by TEM display an amorphous nanostructure of RuS_2_ on RGO crystallized flakes. The specific capacitance measured in planar configuration in 1 M NaCl electrolyte at 5 mV s^−1^ is 238 F g^−1^. This supercapacitor electrode also exhibits perfect cyclic stability without loss of the specific capacitance after 15,000 cycles. In summary, the RGO/Ruthenium active species hybrid material demonstrates remarkable properties for use as active material for supercapacitor applications.

## 1. Introduction

Recently, the need for energy storage systems has become crucial for smart energy management. Supercapacitors are electrochemical power storage devices that can have many practical applications, replacing or working alongside existing battery technology. The main advantages of supercapacitors compared to batteries are their higher power density values, their much longer cycle life, and their fast charge/discharge rate. The fabrication of nanostructured electrodes improves their energy density without affecting their high power density [1,2].

As is well-known, supercapacitors can be divided into two main classes based on different energy storage mechanisms: Electrochemical Double-Layer Capacitors (EDLCs) and Pseudo-Capacitors (PCs). In EDLCs, the storage is based on the adsorption of both anions and cations at the interface between a high surface area electrode and an electrolyte. Typically, porous carbon materials are investigated as electrodes due to their high specific surface area, electrical conductivity and pore size and distribution, which are the main properties. In particular, a self-assembled graphene-based three-dimensional nanoarchitecture aerogel has already been studied by the scientific community and attracted our attention because of its outstanding performance [3,4,5,6,7,8]. As far as concerns PCs, which store energy through fast surface redox reactions (Faradaic processes), they were examined to obtain higher specific capacitance. Usually, metal oxides such as RuO_2_ [9,10,11,12,13], MnO_2_ [14,15,16], Co_2_O_3_ [17], NiO [18], SnO_2_ [19], V_2_O_5_ [20,21,22], NiCo_2_O_4_ [23] are used as pseudocapacitors. Numerous experiments have established that RuO_2_ is the material for pseudocapacitors, which offers the best performance due to its highly reversible charge–discharge features, excellent electrical conductivity and ultrahigh pseudocapacitance [24].

In order to increase the energy density of electrodes for supercapacitors, hybrid materials that combine the properties of both EDLCs and PCs have been developed [25]. Basically, a pseudocapacitive material is synthesized together with a graphene-based aerogel [26,27,28,29,30,31]. These hybrid supercapacitors have the advantage of offering an enhancement of the cycling life of the pseudocapacitive material, an increase of the specific capacitance and avoiding the restacking of the graphene flakes due to the presence of the pseudocapacitive material. In particular, a graphene-based aerogel containing RuO_2_ nanoparticles has already been extensively investigated during the past two decades and has demonstrated good performance [24].

More recently, transition metal dichalcogenide (TMD) mono- and multi-layered sheets have generated considerable research interest due to their unusual physical, chemical and electronic properties compared to their bulky counterparts, along with the great affinity with graphene due to their 2D-layered structure [32,33]. Numerous TMDs have been studied in depth in the literature, for instance MoS_2_ [34,35,36,37,38], TiS_2_ [39,40], WS_2_ [36,41], MoSe_2_ [37,38] and WSe_2_ [37,42]. In addition, some of these have been reported as outstanding materials in a hybrid configuration for supercapacitor applications, such as MoS_2_ [43,44,45,46] and WS_2_ [47,48].

Although hybrid supercapacitors containing TMDs have demonstrated better performance, only a limited number of hybrids is reported in the literature [49]. Furthermore, little work has been done on the synthesis of Ruthenium-based TMD, even though it is the transition metal, when combined in oxides, with the best performance as pseudocapacitive material [9,24]. In practice, Ruthenium-based TMD, and especially RuS_2_, can be synthesized by energy and time-consuming processes [50,51,52]. More recently, greener routes have been developed based on hydrothermal synthesis. However, these processes are based on thiourea [53,54], as Sulphur precursor, which is suspected of causing cancer in addition to be harmful for the user, as reported in the Safety Data Sheets (SDS). Thus, it is clear that L-cysteine, a natural amino acid which is already reported for the synthesis of MoS_2_ [55], can be attractive as a green precursor of Sulphur and, for this reason, it has been used in this work. Moreover, to the best of the authors’ knowledge, no work has previously been done on the synthesis of hybrid materials containing RuS_2_. Thus, the novelty of this work deals with the filling of the gap of literature in this field.

This paper focuses on the synthesis of a new hybrid material for electrodes in supercapacitor applications by hydrothermal synthesis. Starting from an aqueous dispersion of graphene oxide (GO), a precursor of Ruthenium (RuCl_3_) and L-cysteine as the precursor of Sulphur, we demonstrate an easy way to obtain an aerogel of reduced graphene oxide (RGO) decorated with Ruthenium active species containing Ruthenium Sulfide (RuS_2_). This TMD is able to enhance the specific capacitance by Faradaic processes thanks to its structural properties. As this hybrid material has never been synthesized before, a full characterization was required. For this purpose, we performed a careful examination by Field Emission Scanning Electron Microscopy (FESEM), Transmission Electron Microcopy (TEM), Energy-Dispersive X-ray spectroscopy (EDX), X-ray Diffraction (XRD), X-ray Photoelectron Specteoscopy (XPS) and micro-Raman, in addition to electrochemical characterization in planar configuration carried out by Cyclic Voltammetry and Galvanostatic Charge/Discharge measurements.

## 2. Results and Discussion

### 2.1. Characterization of Morphology and Structure

**Figure 1:**
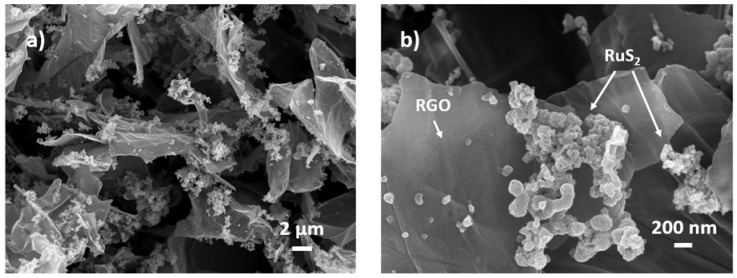
Low-magnification (**a**) and high magnification (**b**) FESEM images of RGO aerogel decorated with Ruthenium active species containing RuS_2_ nanostructures.

The morphology of a typical aerogel sample is presented in Figure 1. It is clear from low magnification images (See Figure 1a) that the sample exhibits a three-dimensional structure of interconnected RGO flakes, which forms during the hydrothermal treatment, in accordance with results previously reported in literature [56]. The slightly wrinkled flakes have lateral size of micrometer order and they are decorated with nanostructures. High resolution images (See Figure 1b) show that these nanostructures are almost spherical (with diameter in the 5–50 nm range) and they are distributed in sub-micrometer agglomerates inside the aerogel. EDX analysis (shown in the Supporting Information) was used as preliminary confirmation of the successful synthesis of Ruthenium active species nanostructures: in fact, the detected elements are C, O, Ru, S and Cl. The presence of Cl is due to the RuCl_3_ synthesis precursor unreacted and physisorbed on the surface.

The overall morphology of the aerogel is promising for supercapacitor application: in fact, the porous 3D structure leads to facile impregnation with the electrolyte. Moreover, the successful decoration of the RGO flakes with Ruthenium active species, and in particular RuS_2_ nanoparticles, can in principle boost the capacitance of the electrode through Faradaic reactions at the surface of the nanoparticles. A more refined overview of the chemical composition of the material was obtained by XPS spectroscopy (Caltech, Pasadena, CA, USA). It is well known that this spectroscopy technique allows for the investigation of the chemical state of detected elements and therefore it is often performed for chemical phase identification [57]. Moreover, since the sampling depth of XPS is <10 nm, this technique is particularly well-suited for the analysis of materials in view of their application in devices that rely on surface effects.

**Figure 2:**
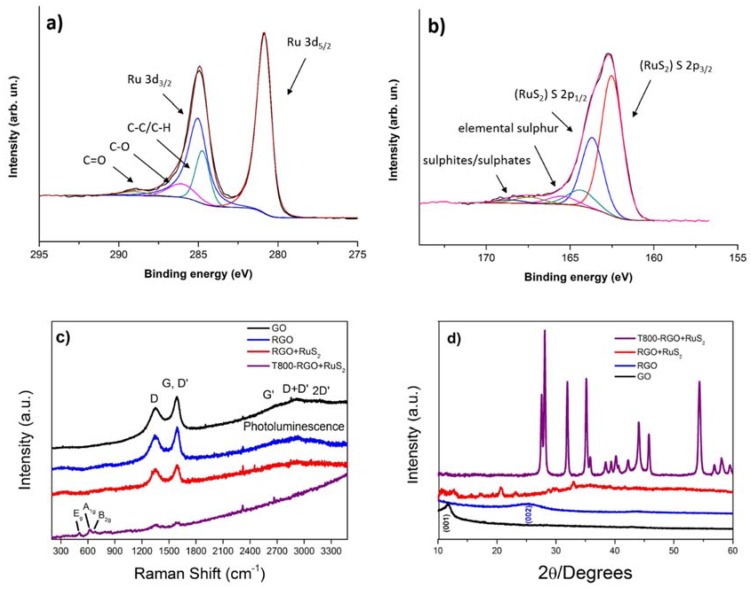
XPS High resolution scans of (**a**) C1s/Ru3d and (**b**) S2p regions of RGO aerogel decorated with Ruthenium active species containing RuS_2_ nanoparticles; (**c**) Raman spectra of pristine GO, RGO, RGO/Ruthenium active species (before and after thermal annealing in inert gas) aerogels; (**d**) XRD of pristine GO, RGO and as-synthesized RGO/RuS_2_ (before and after thermal annealing in inert gas).

At first, a survey spectrum (see Supplementary Information) was acquired, confirming the presence of the chemical elements previously outlined by EDX and highlighting traces of N as probable elemental residue in the starting commercial GO flakes, as a consequence of the oxidation process of graphite. High Resolution (HR) scans of the most significant regions of the photoelectron spectrum were acquired and they are shown in Figure 2a,b. Concerning carbon, the dominant contribution to the C1s/Ru3d region of the C–C peak at 284.8 eV with respect to peaks associated to carbon–oxygen bonds proves the successful reduction of GO. Regarding the Ru3d doublet, the positions of the two peaks (280.8 eV, 285.0 eV) are compatible with RuS_2_ phase, according to the literature [53,58]. Further confirmation of the presence of RuS_2_ is provided by the Ru3p_3/2_ peak (462.4 eV, shown in Supporting Information) and by the S2p doublet (162.5 eV, 163.7 eV), which all approximately agree with previous XPS analyses [59]. The S2p region is characterized by two more doublets at higher binding energies, which can be related to elemental Sulphur [60] and sulphites/sulphates [61]. As previously stated, both EDX and XPS survey analysis point to the presence of Chlorine, which was attributed to a residue of the RuCl_3_ precursor. Further confirmation is given by the deconvolution of the Cl2p region of the photoelectron spectrum (Supporting Information), with binding energies (Cl2p_3/2_ = 197.9 eV, Cl2p_1/2_ = 199.5 eV), which, according to the literature [62], can be possibly ascribed to RuCl_3_, although it has been recently proposed that this doublet can be assigned to HCl [63].

It was possible to calculate relative atomic concentration values for Ru, S and Cl from Ru3p_3/2_, S2p and Cl2p regions, respectively. The Ru3p_3/2_ region was chosen for Ru since it does not overlap with other elements. It is interesting to note that the atomic concentration ratio S/Ru is 2.25, while when we consider just the S2p doublet assigned to RuS_2_ phase, the ratio is S_RuS2_/Ru ≈ 1.85. These results provide proof that RuS_2_ phase is obtained in the Ruthenium active species, with acceptable stoichiometry. Finally, it must be stressed that the Cl/Ru ratio is 0.08, therefore making the RuCl_3_ residue concentration negligible with respect to the dominant RuS_2_ phase.

Raman spectra, reported in Figure 2c, depicts how the hydrothermal process is efficient in the reduction of the pristine GO to RGO even in the presence of the Ru and S precursors, as demonstrated by the increase of the I_D_/I_G_ ratio (from 0.78 to 0.98) that suggests the formation of more sp^2^ domains [64] and the decrease of photoluminescence ascribed in graphene oxide to band-gap emission from electron-confined sp^2^ islands and to oxygen-related defect states [65]. In addition to the typical Raman peaks of RGO [65], for the RGO/Ruthenium active species hybrid aerogel, a slight broadening of the G peak at 1589 cm^−1^ is observed together with a loss of bands resolution in the 2600–3200 cm^−1^ range, maybe due to interference phenomena, in sp^2^ cluster stacking, because of the formation of RuS_2_ structures. Unfortunately, the related spectrum (red line) does not give evidence of vibrational modes assignable to Ru-S containing species, as expected in the 350–450 cm^−1^ range [66]. Only when the aerogel is annealed at 800 °C, for 2 h, under inert gas flux, it is possible to single out (purple line) at 504, 615 and 685 cm^−1^ the three RuO_2_ main features assigned to E_g_, A_1g_ and B_2g_ modes, respectively [67]. This result is in accordance with XRD characterization (discussed in the following), so confirming that the RuS_2_ contained in the hybrid aerogel is scarcely crystalline, and, only thanks to the annealing process, it is possible to obtain a crystalline, but unstable, material that, in contact with atmospheric oxygen, tends to oxidize. As Raman spectroscopy is a surface investigation technique, the effect of environmental oxidation is more evident than for X-ray Diffraction, which is a bulk investigation technique.

Figure 2d reports the XRD pattern on the RGO/Ruthenium active species (containing RuS_2_ nanoparticles sample) synthesized by hydrothermal method. The diffraction peaks are very weak, indicating that both RGO and RuS_2_ arrange in short range ordered structure. This fact was already observed for MoS_2_ flakes [46,55]. Moreover, the amorphous (quite disordered) structure of the Ruthenium Sulphide is due to the low temperature process. Indeed, the Ruthenium Sulphide starts to crystallize at a temperature of 650 °C as reported previously [54]. Nevertheless, the three peaks with the higher intensity (red line) correspond to the elemental Sulphur (20.47° and 22.97°) and RuS_2_ (32.73°), respectively. In addition, all of the other peaks match with the ones of the reference spectra of RuS_2_ (JCPDS card N°00-019-1107); RuO_2_ (JCPDS card N°00-040-1290); Ru (JCPDS card N°00-006-0663); RuCl_3_ (JCPDS card N°00-036-1225) and S (JCPDS card N°00-023-0562). In order to increase the crystallinity, an annealing in inert atmosphere for 2 h at 800 °C was carried out. The XRD pattern of the annealed sample (purple line) is reported in Figure 2d. The crystallinity has been increased by the annealing process. All of the typical peaks of the Ruthenium Sulphide are displayed and confirm the presence of the dichalcogenide structures in the as-prepared material synthesized by hydrothermal method. However, peaks corresponding to RuO_2_ and Ru can also be observed. Furthermore, the disappearance of RuCl_3_ and S can be attributed to a desorption of the unreacted species on the surface of the hybrid material.

**Figure 3:**
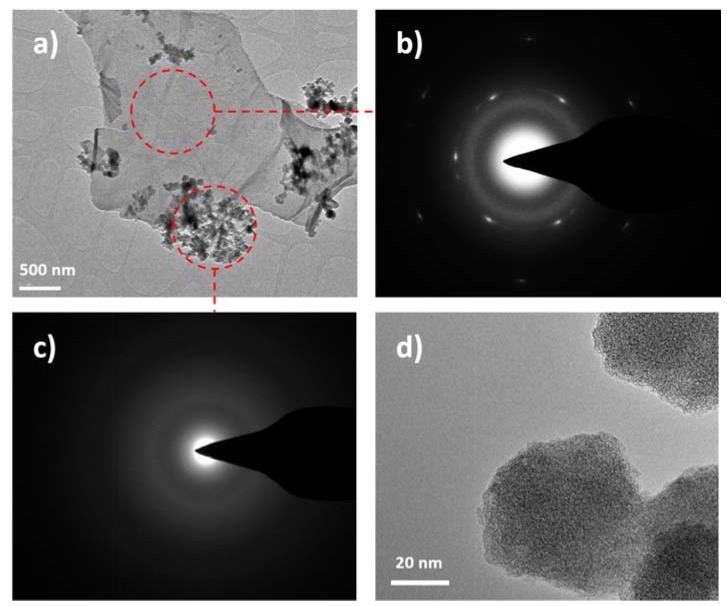
(**a**) low-magnification Bright Field TEM image of RGO flake decorated with Ruthenium active species nanoparticles. SAED pattern of RGO flake (**b**) and Ruthenium active species nanoparticles (**c**); High Resolution TEM image of Ruthenium active species nanoparticle (**d**).

TEM (FEI, Hillsboro, OR, USA) characterization was conducted on the aerogel in order to reach deeper insight into the morphology and crystalline structure of the sample. Figure 3a shows a low magnification bright field image of a typical RGO flake decorated with RuS_2_ nanostructures, which are clearly visible as low-intensity features due to mass contrast. A selected area electron diffraction (SAED) pattern of a clear region of the RGO flake (Figure 3b) confirms the characteristic in-plane hexagonal ordering of carbon atoms typical of graphene. This is a further indication that the reduction process under hydrothermal treatment allows for good in-plane arrangement of carbon atoms. This spatial configuration assures a good electrical conductivity between the 3D structures of interconnected RGO. On the other hand, SAED patterns obtained on agglomerates of RuS_2_ nanoparticles (Figure 3c) suggest amorphicity, or at least low crystalline quality. This is further confirmed by high resolution images (such as Figure 3d), where no lattice fringes are visible.

### 2.2. Electrochemical Performance

**Figure 4:**
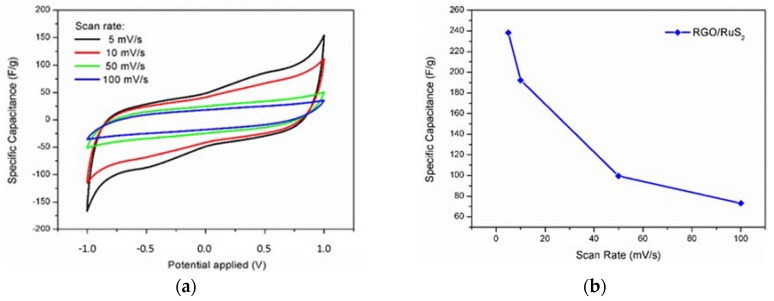
(**a**) cyclic Voltammograms of RGO/Ruthenium active species hybrid at different scan rate; (**b**) evolution of the Specific Capacitance with the scan rate.

Hybrid materials synthesized by using the hydrothermal method were investigated by Cyclic Voltammetry (CV) in a two-electrode configuration and a potential window of 2 V. Results obtained at different scan rates are shown in Figure 4a. The box-like voltammogram indicates a typical double layer capacitor behavior. The signals due to pseudo-capacitance derived from oxygen-containing functional group are weak, which is in perfect agreement with XPS and Raman analyses. In addition, the pseudo-capacitance effects related to the Ruthenium active species, and in particular RuS_2_, in addition to nanostructures are not clearly distinguishable probably due to the low concentration in the hybrid. The evolution of the Specific Capacitance as a function of the scan rate is reported on Figure 4b. The storage ability of the hybrid is almost five times higher than the one observed for pure RGO (191 vs. 33 F/g [46], at 10 mV/s). Moreover, the highest RGO/Ruthenium active species Specific Capacitance was obtained at 5 mV/s and corresponds to a value of 238 F/g. This result is remarkable for a green one-pot hydrothermal synthesis.

**Figure 5:**
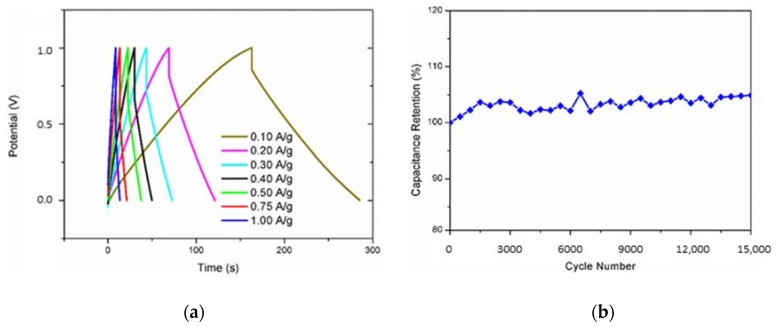
(**a**) charge–discharge curves at different current density and (**b**) evolution of the capacitance retention in function of the cycle number for the RGO/Ruthenium active species hybrid.

Galvanostatic Charge/Discharge measurements were performed on the hybrid sample (see Figure 5a). The potential window was reduced to 1 V to avoid the water splitting effects. Galvanostatic charge–discharge curves reveal that all the materials own good capacitive behavior due to the linearity of the chronopotentiograms. The ohmic resistance (depicted by the voltage drop (IR drop) in curves of Figure 5a) is probably due to the presence of sulphites/sulphates (SO_x_) species. Finally, and very interestingly, RGO/Ruthenium active species hybrid materials demonstrate a perfect stability after 15,000 cycles (see Figure 5b). Indeed, it is possible to observe a steady increase in capacitance (about the 5%) for the hybrid. As reported in our previous work, the reference (RGO) has a lower stability with almost 95% of the capacitance retained after the same number of cycles [46]. The higher stability of the hybrid material is the result of a surface activation effect [68] and an increased porosity of the carbonaceous matrix that allows rapid diffusion of the electrolyte through the porous structure [69].

## 3. Materials and Methods

### 3.1. Materials

Graphene Oxide (Single Layer, thickness: 0.7–1.2 nm, purity 99 wt %) was purchased from Cheaptubes (Cambridgeport, VT, USA). L-cysteine used as precursor of Sulphur and RuCl_3_ used as precursor of Ruthenium were purchased from Sigma Aldrich (Milano, Italy) and Alfa Aesar (Heysham, UK), respectively. NaCl (anhydrous, ACS reagent, purity ≥ 99.9%, Sigma Aldrich, Milano, Italy) was used as electrolyte. Poly(vinylidene fluoride) was used as binder after dispersion in Dimethyl Sulfoxide (DMSO, purity ≥99.5% (GC), Sigma (Milano, Italy)) Carbon Black (purity ≥ 99.9%, Alfa Aesar (Heysham, UK)) was used as conductive material in the electrode fabrication. All products were used without purification.

### 3.2. Preparation of RGO, RGO/Ruthenium Active Species Aerogels

Graphene oxide (GO) was dispersed in de-ionized water (2 mg/mL for 17 mL total volume) at room temperature and sonicated for 30 min. After sonication, the brown solution was transferred in a Teflon lined stainless steel autoclave with total volume of 26 mL and heated at a temperature of 180 °C for 12 h, in a muffle furnace. After the hydrothermal reaction, the autoclave was cooled naturally until room temperature. Then, the black hydrogel obtained was rapidly frozen using liquid nitrogen and dried overnight under vacuum to obtain the reduced graphene oxide (RGO) aerogel. For the RGO/Ruthenium active species sample, 132.8 mg of RuCl_3_ as Ruthenium precursor and 61.5 mg of L-cysteine were added to the GO in order to obtain a Ru/S atomic ratio equal to 1/3. Materials were dispersed in de-ionized water at room temperature and the same procedure used for RGO aerogel was repeated.

### 3.3. Materials Characterizations

The morphology and composition of the synthesized products were characterized before the tests in supercapacitor configuration.

The morphology of the aerogel samples was characterized by means of Field Emission Scanning Electron Microscopy (FESEM) with a Zeiss Supra 40 microscope (Zeiss, Milano, Italy) equipped with a Oxford Instruments 10 mm^2^ Si(Li) Energy Dispersive X-ray (EDX) spectrometer (X-Max^N^, Abingdon-on-Thames, UK) for elemental analysis.

The crystallinity of the hybrid material was evaluated by X-ray Diffraction by using a PANalytical X’Pert Powder (Cu Kα radiation, Lissone, Italy); 2θ range: 10–80; 2θ step: 0.028; step time: 2 s, with fixed specimen and moving X-ray source and detector simultaneously of a θ angle.

Raman spectroscopy on aerogels were performed in order to study the extent of reduction of GO and the effect of hybrid composition on the samples vibrational features. Hybrid materials were characterized, in backscattering light collection through a microscope objective (50X) by means of a Renishaw InVia Reflex micro-Raman spectrometer (Renishaw plc, Sheffield, UK), equipped with a cooled charge-coupled device (CCD) camera and a 514.5 nm diode laser (selected excitation wavelength). Each spectrum was collected after gently pressing the aerogel powder on a microscope glass slide, with the following parameters: 10 mW laser power, 10 s of exposure time and 1 accumulation.

Further insight into the morphology and crystalline structure of the samples was obtained by Transmission Electron Microscopy (TEM), using an FEI Tecnai G2 F20 S-TWIN instrument (FEI, Hillsboro, OR, USA) operated at 200 kV acceleration voltage. Concerning sample preparation for TEM analysis, a drop of aerogel dispersion in ethanol was deposited on a holey carbon copper grid. Gatan Microscopy Suite software (GMS 3, Gaitan, Abingdon, UK) was used for the processing of high-resolution images and electron diffraction patterns.

The chemical composition was investigated with a PHI 5000 Versaprobe scanning X-ray photoelectron spectrometer (Phi, Roma, Italy) (monochromatic Al K-alpha X-ray source with 1486.6 eV energy). Concerning the detection of the photoelectron signal, different pass energy values were employed: 187.85 eV for survey spectra and 23.5 eV for high-resolution peaks. Spectra were analyzed using MultiPak 9.6 (Phi, Roma, Italy) and CasaXPS softwares (Caltech, Pasadena, CA, USA). Charge neutralization during measurements was obtained with a combined electron and Ar^+^ ion gun neutralizer system. All core-level peak energies were referenced to C1s peak at 284.8 eV (C–C/C–H bonds) after fitting the C1s/Ru3d region with suitable synthetic components. The background contribution in HR spectra was subtracted by means of a Shirley function [70].

### 3.4. Electrodes Preparation

For all the aerogel materials, the electrode slurry was composed by a ratio of active synthesized aerogel/carbon black/PVDF (Polyvinylidene Difluoride) equivalent to 15/1.5/1. PVDF was used as binder and carbon black was used to assure electrical contact between the flakes of RGO in the three-dimensional nanostructure.

### 3.5. Supercapacitor Device and Electrochemical Measurements

A thin film about 50 nm of Platinum was deposited by Direct Current sputtering on two microscopes slides. These glasses were used as current collectors. The devices were fabricated in symmetric electrodes configuration and were sealed by means of a thermoplastic thin film polymer. Clean room paper was used as separator. Electrolyte, 1 M NaCl, was inserted in the dispositive by means of a depression created by a vacuum pump. The Cyclic voltammetry measurements were performed with a Metrohm Autolab PGSTAT128 potentiostat/galvanostat (Metrohm, Barendrecht, The Netherlands). Galvanostatic charge/discharge measurements were obtained with an Arbin Instrument Testing System model BT-2000 (Arbin Instruments, College Station, TX, USA). Both kinds of measurements were performed in device configuration (e.g., two symmetric electrodes).

### 3.6. Calculation of Capacitance

Specific capacitance for the supercapacitors was calculated from the cyclic voltammograms measurements according to the method established by Stoller and Ruoff for one electrode [71]:

In voltammetry, the capacitance is defined as the area under the current transient during one linear scan of the voltammogram normalized with respect to the employed potential window (ΔV), as:

(1) $$C=\frac{\int_{t_{1}}^{t_{2}}i\;\mathrm{dt}}{\Delta V}$$

The specific capacitance of one electrode can be estimated for a two-electrode cell and in device configuration by the following equation:

(2) $$C_{S}=4\;\frac{C}{m_{1}+m_{2}}$$

where m_1_ and m_2_ are the masses of the first and the second electrodes and the multiplier of four adjusts the capacitance of the cell and the combined mass of two electrodes to the capacitance and mass of a single electrode.

## 4. Conclusions

In this paper, we report an easy and green one-pot hydrothermal synthesis of an RGO/Ruthenium active species (containing RuS_2_ nanoparticles) hybrid for electrodes in supercapacitor applications. The synthesis is carried out in a Teflon lined stainless steel autoclave from Graphene Oxide, Ruthenium Chloride and L-cysteine, an amino acid, as Ruthenium and Sulphur precursor, respectively. The low temperature process (180 °C) allows for synthesizing Ruthenium active species containing RuS_2_ nanostructures supported on reduced graphene oxide flakes (RGO), for which interconnected porous structure is a key feature for electrodes in supercapacitor applications because of the promotion of electrolyte diffusion throughout the pores and, indeed, the increase of the electrode/electrolyte interface.

FESEM analysis displays a three-dimensional structure of interconnected RGO flakes decorated with nanostructures and XPS characterization confirms the RuS_2_ composition: the successful reduction of graphene oxide in RGO and the reached RuS_2_ proper stoichiometry. XRD patterns highlight that both RGO and RuS_2_ arrange in short range ordered structure, due to the Ru/S precursors low ratio, chosen for the hydrothermal synthesis. After annealing in inert atmosphere, only, the observed diffraction peaks correspond to the Ruthenium active species containing RuS_2_ crystalline nanostructure. Moreover, Raman analysis confirms that Graphene Oxide is successfully reduced and TEM images display RGO crystalline flakes decorated with Ruthenium active species containing RuS_2_ amorphous nanostructures. The SAED (Selected Area Electron Diffraction) patterns show the characteristic in-plane hexagonal ordering of carbon atoms typical of graphene and the amorphous state or, at least, a poor crystallinity of RuS_2_ nanostructures.

Finally, by means of electrochemical characterization, a noticeable Specific Capacitance of 238 F/g, at 5 mV/s, is reached by using the RGO/Ruthenium active species containing RuS_2_ nanoparticles hybrid as electrode material and a relevant stability, over 15,000 cycles (current density: 1 A/g), is obtained. These two achievements suggest that the hybrid structure exhibits the potentiality for exploitation as electrode component in supercapacitors.

## Supplementary Materials

The following are available online at www.mdpi.com/1996-1944/11/1/57/s1. Representative EDX spectrum of RGO/Ruthenium active species sample, Figure S2: Survey spectrum of RGO sample decorated with RuS_2_ nanostructures, Figure S3: High-resolution XPS scans of the Ru3p_3/2_ (a) and Cl2p (b) regions.
