# Supplementary material for: Graphene/Ruthenium Active Species Aerogel as Electrode for Supercapacitor Applications
Source: Materials (Basel). 2017 Dec 30;11(1):57. doi: 10.3390/ma11010057 (PMC5793555; doi:10.3390/ma11010057)
Supplement: Supplementary file 1 [file materials-11-00057-s001.pdf]

Supporting Information

Graphene/Ruthenium Sulphide aerogel as electrode for supercapacitor applications

Arnaud Gigot [1,2,*], Marco Fontana [2], Candido Fabrizio Pirri [1,2] and Paola Rivolo [2]

1 Dipartimento di Scienza Applicata e Tecnologia, Politecnico di Torino, 10129, Torino, Italy
2 Center for Sustainable Future Tecnologies, Istituto Italiano di Tecnologia, 10129, Torino, Italy
* Corresponding Author: arnaud.gigot@polito.it; Tel: +39 011 090 7343

EDX ANALYSIS

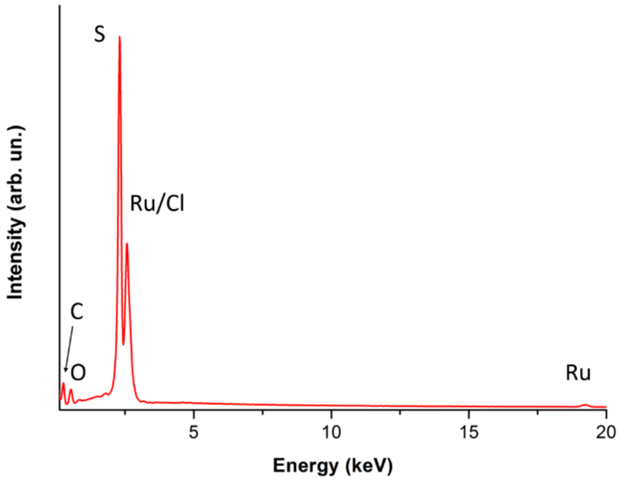

Figure S1: Representative EDX spectrum of RGO/RuS_2 sample.

XPS ANALYSIS

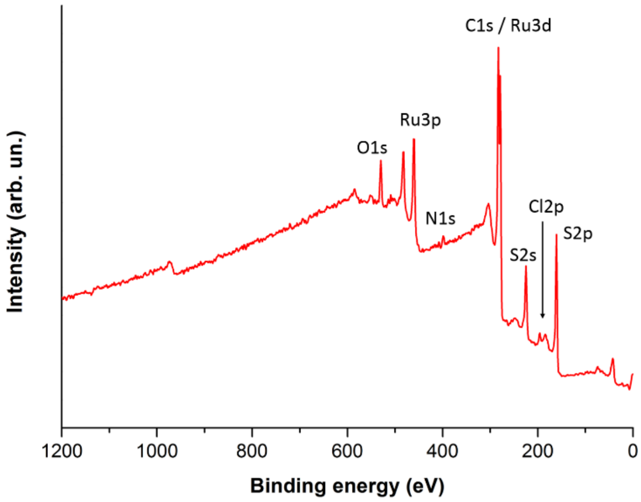

Figure S2: Survey spectrum of RGO sample decorated with RuS_2 nanostructures.

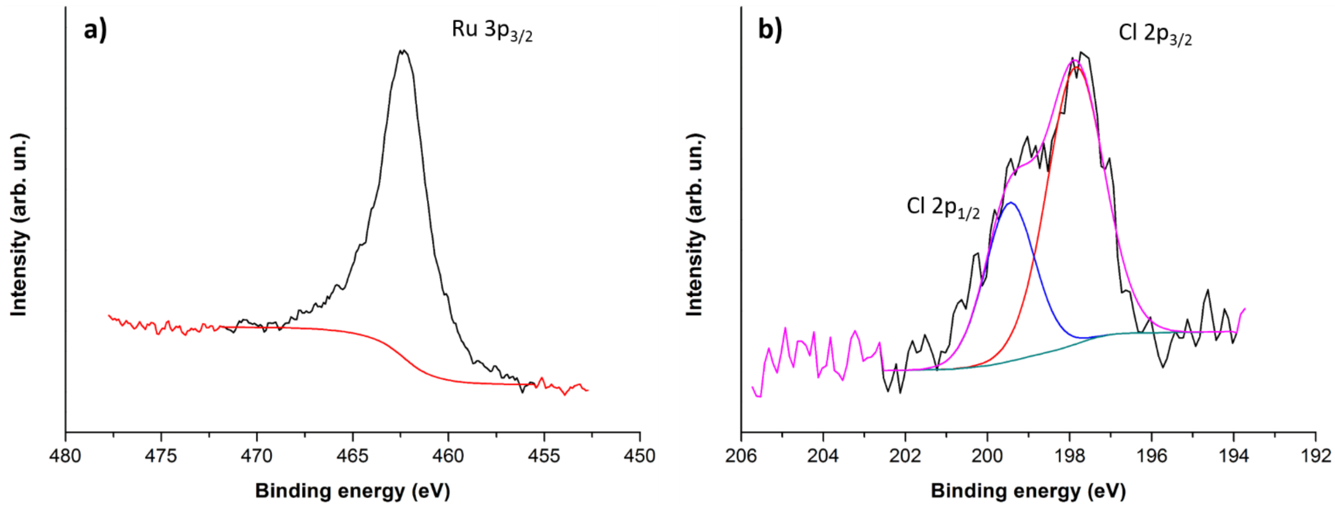

Figure S3: High-resolution XPS scans of the Ru $3p_{3/2}$ (**a**) and Cl 2p (**b**) regions.
